# Virtual Versus Traditional Learning: A Comparison of Dental Students’ Perception and Satisfaction

**DOI:** 10.3390/dj12120393

**Published:** 2024-12-04

**Authors:** Marwa Madi, Balgis Gaffar, Faraz Ahmed Farooqi, Osama Zakaria, Shazia Sadaf, Muhanad Alhareky, Jehan AlHumaid

**Affiliations:** 1 Department of Preventive Dental Sciences, College of Dentistry, Imam Abdulrahman Bin Faisal University, P.O. Box 1982, Dammam 31441, Saudi Arabia; bgosman@iau.edu.sa (B.G.); malhareky@iau.edu.sa (M.A.); jaalhumaid@iau.edu.sa (J.A.); 2 Department of Dental Education, College of Dentistry, Imam Abdulrahman Bin Faisal University, P.O. Box 1982, Dammam 31441, Saudi Arabia; fafarooqi@iau.edu.sa (F.A.F.); ssahmad@iau.edu.sa (S.S.); 3 Department of Biomedical Dental Sciences, College of Dentistry, Imam Abdulrahman Bin Faisal University, P.O. Box 1982, Dammam 31441, Saudi Arabia; oazakaria@iau.edu.sa

**Keywords:** virtual learning, traditional learning, perceptions, dental students, satisfaction

## Abstract

**Background:** Comparing the effectiveness of virtual and traditional learning methods is crucial for understanding their impact on knowledge transmission in different subjects. The objective of this cross-sectional study was to explore dental students’ perceptions and satisfaction levels with their experiences in virtual learning compared to traditional classroom learning. **Methods:** A cross-sectional survey was administered to all dental students in the College of Dentistry across preclinical (3rd and 4th year) and clinical (5th and 6th year) levels using Google Forms. The questionnaire included items from the validated student survey component of the Blended Learning Toolkit, the Quality Matters Higher Education Rubric, and the Web Learning Project Student Survey. **Results:** With a 93% response rate from 313 students, the survey results indicated a strong preference (87%) for traditional learning among 3rd-year preclinical students who favored it over virtual learning, contrasting with 54% of 6th-year clinical year students. Most of the students acknowledged that traditional learning facilitates the use of various instructional methods while virtual learning hinders interactions among each other (*p* = 0.068). However, virtual lectures were perceived as more organized (70% to 89%, *p* = 0.014). Gender differences were minimal in preference for virtual learning, but male students significantly preferred traditional methods (*p* = 0.001). **Conclusion:** The results indicate a preference for traditional over virtual learning, especially among males and preclinical dental students, emphasizing the need for an integrated approach that combines the structured benefits of virtual learning with the interactive advantages of traditional classrooms.

## 1. Introduction

Effective education, characterized by independent, guided, self-directed learning, is fundamental to student success and knowledge development [1]. While traditional learning involves classroom-based instruction with direct mentor presence [2], long-lasting student interactions and communication are crucial educational components [3]. Virtual learning utilizes learning management systems (LMSs), incorporating various tools like learning paths, quizzes, and interactive platforms for student–mentor engagement [4].

Contemporary evidence suggests that students prefer challenging and dynamic educational environments [1,5,6], necessitating a balance between student needs and educational system capabilities [5].

Studies comparing learning modalities have revealed contrasting findings [5,6,7]. In nursing education, traditional learning was found to be exhausting, with its time constraints potentially hindering effective learning, while virtual learning’s flexibility in terms of content access enhanced instructional effectiveness [8]. Similarly, in medical education, students showed a preference for web-based tutorials over traditional lectures, citing advantages such as accessibility, navigational freedom, high-quality visual content, and opportunity for repeated practice [9].

Virtual and traditional learning have differences in terms of efficacy and knowledge transmission, and it is imperative to assess and compare the efficacy of these methods for the instruction of different topics [10,11]. Electronic learning programs have been used to teach and learn various topics in dentistry [11]. Previous studies have evaluated the efficacy of virtual instruction of topics in oral and maxillofacial radiology. They reported that students’ performance and knowledge had improved significantly, suggesting merging e-learning and traditional teaching to enhance students’ experience [12,13,14].

During traditional learning, students are able to interact with their tutor and peers during collaborative, hands-on activities and feedback, which enrich their learning experience and improve their understanding of essential concepts [15]. This can be explained by the social constructivist theory [16], which emphasizes the role of interaction and collaboration of the student with his peers and tutors for stimulating effective learning [15].

Some researchers believe that the flexibility of online learning makes it interesting to students [17,18], and there is evidence that this influenced the perceived usefulness of online learning technology and, subsequently, generally positive attitudes toward this technology [19,20]. Faculty and students’ abilities to use technology, as well as their motivation in online learning, can affect learning success [19,21,22]. Students similarly need to change how they perceive learning through the use of multiple online tools and strategies.

In IT service delivery, satisfaction represents the emotional response to needs fulfillment [15]. This concept was applied for online learning transition during the COVID-19 pandemic, when satisfaction reflected students’ emotional responses to the effectiveness of online education as a sociotechnical system [16]. Studies suggest that elements influencing online learning satisfaction correlate with students’ perceptions of their learning success [16,17,18].

During and following the COVID-19 crisis, dental schools have increasingly implemented virtual teaching methods for various educational activities, including lectures, assessments, and presentations. In light of this significant shift to online platforms, this study aimed to evaluate dental students’ perceptions and satisfaction with virtual teaching as compared to traditional teaching methods. The null hypothesis was that there would be no significant difference between traditional and virtual learning with regard to theoretical knowledge, perception, and student satisfaction.

This assessment seeks to better understand how well virtual teaching has served educational needs during emergencies and what improvements might be needed if it continues to be a substantial component of dental education.

## 2. Materials and Methods

### 2.1. Study Design and Study Participants

This cross-sectional survey-based study was conducted in the College of Dentistry at Imam Abdulrahman Bin Faisal University after obtaining approval from the Institutional Review Board (IRB-2024-01-196). All dental students registered in preclinical levels (3rd and 4th years) and clinical levels (5th and 6th years) were invited to participate in this study. First and second year dental students were excluded from the study due to the nature of the foundational and laboratory-based courses that they receive.

An informative text was inserted at the top of the questionnaire that explained the nature of the study; students stating their agreement to participate in the study had to select this written informed consent. Those who agreed to participate were provided access to the following pages of the survey, and their answers were included in the study. Anonymous responses in the questionnaire were used to ensure confidentiality and privacy of the respondents. No personal information (name, College ID number, or any other type of information that allows for the identification of students) was obtained from the students in the questionnaire. Only the research team had access to all the data collected.

### 2.2. Data Collection Tool and Procedure

A questionnaire was developed to assess students’ perceptions regarding virtual learning compared to the traditional learning methods. The finalized questionnaire contained 18 closed-ended questions that were divided into two parts. The first part collected information about the students’ gender, study year, and previous exposure to instructors. The second part included questions about students’ perceptions of virtual and traditional learning. Some of the items of the questionnaire were adopted from the validated student survey component of the Blended Learning Toolkit developed by the University of Central Florida and the American Association of State Colleges and Universities [23]. Other items were adopted from the standardized distance education quality metrics [24] and the Web Learning Project Student Survey [25]. The questionnaire was validated and pilot-tested to ensure its reliability and validity. The questionnaire was uploaded online on the survey platform “GOOGLE SURVEY” (surveys.google.com, Google LLC, Mountain View, CA, USA), and the link was sent to the students’ institutional emails. Students who consented to participate were informed about the study and asked to complete the online questionnaire.

### 2.3. Statistical Analysis

Study variables included gender (male or female), year of study (third, fourth, fifth, sixth), and previous exposure to course instructors (yes, all of them; yes, some of them; no). The study exposure was the type of class (traditional or virtual), and the study outcomes were students’ satisfaction and perception.

In the process of analyzing survey data, a systematic method was employed to translate responses into quantifiable scores. This involved assigning numerical values to each response category, such as assigning a score of 2 for “Yes,” 1 for “No,” and 0 for “no difference”. These scores were then aggregated to compute a total score for each respondent. Survey data were downloaded from Google Forms, and an Excel spreadsheet was generated; data were coded and transferred to SPSS version 22 (IBM Corp., Armonk, NY, USA). Categorical data were expressed as frequency and percentages, while continuous variables were expressed as mean and standard deviation (+SD). The Chi-square test was used for comparing proportions. Student’s *t*-test/ANOVA was used for comparing means among groups. Statistical significance was set to 0.05 or less, and a confidence interval of 95%.

## 3. Results

A total of 313 undergraduate dental students (93% response rate) participated in this study with nearly equal representation from both genders: 154 male (49%) and 159 female (51%) students. The distribution across academic years was also balanced, as 30% of students were in their 3rd and 4th years, and 20% were in their 5th and 6th years. Forty-two percent of students had exposure to their course instructor for the first time, followed by 37% who were exposed to some of the teachers already. The majority of students (52%) expressed a preference for traditional classroom learning over virtual learning, citing reasons such as increased interaction (45%), diverse teaching styles (61%), a better understanding of course material (54%), and difficulty being attentive all the time in a virtual classroom (59%). Interestingly, 62% believed that virtual classrooms were more effective for preclinical courses, having control over the pace of learning (58%) and improving time management (51%), while fewer students were optimistic about virtual assessments (33%) and grades (26%) (Table 1). It is noteworthy that a majority of students reported that factors, such as lecture outlines and grade distribution, did not affect their preference between virtual and traditional learning, with 51% and 41% indicating so, respectively.

The questionnaire items were categorized based on their relevance to perceptions of online/virtual and traditional learning. In Table 2, the students’ perceptions of virtual learning were presented, revealing significant differences between preclinical and clinical years’ students. Preclinical year students, constituting 82%, mostly expressed disfavor toward virtual learning, contrasting with the clinical students’ satisfaction (*p* = 0.001). Notably, while 6th-year students leaned towards the belief that virtual classes could enhance management skills, the majority of 3rd-year students disagreed with this concept (68% vs. 63%). Furthermore, most participants believed that virtual settings were suitable for preclinical courses, although this sentiment was not shared by 4th-year students. Additionally, a majority of preclinical students responded “No” to the virtual OSPE (Objective Structured Preclinical Examination), in contrast to 5th- and 6th-year students (63% vs. 47%, respectively). Overall, the perceptions regarding virtual learning varied significantly between preclinical and clinical students, with the majority of items in Table 2 demonstrating significant differences.

**Figure 1:**
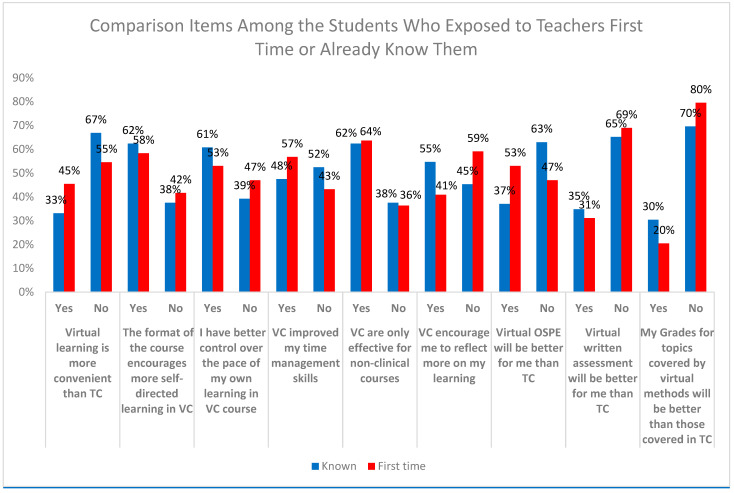
Comparison of preferences for virtual learning items between students meeting instructors for the first time and those already acquainted with them.

Figure 1 shows the comparison of virtual learning items’ preference among those who were exposed to the course instructors for the first time or were already known to them. Students who have prior familiarity with their instructors from previous classes were more supportive of virtual learning compared to those encountering their instructors for the first time. This preference may stem from the established relations and trust between students and instructors, making virtual interactions more comfortable and familiar.

In Table 3, students’ responses regarding the traditional learning approach were presented, showing differences between preclinical and clinical students. Among preclinical students (3rd and 4th years), there was a significantly higher overall preference for traditional learning over virtual learning, 70% and 58%, respectively, compared to clinical students (*p* = 0.001). Regarding interactions, all students, regardless of academic year, generally agreed that virtual learning does not allow for interaction between students (*p* = 0.068). Most students across all academic years acknowledged that traditional learning facilitates the use of various instructional methods. However, there was a perception among the majority of students that virtual lectures are more organized compared to traditional ones, with percentages ranging from 70% to 89% (*p* = 0.014). Preclinical students (3rd and 4th years) expressed challenges in maintaining attentiveness throughout virtual classes, (73% vs. 57%, respectively) (*p* < 0.001).

**Figure 2:**
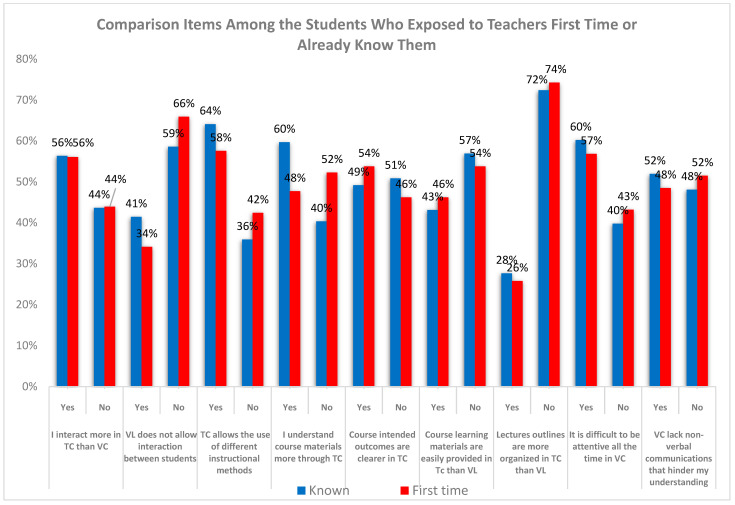
Comparison of preferences for traditional learning items between students meeting instructors for the first time and those already acquainted with them.

Figure 2 presents a comparison of preferences for traditional learning items between students who were meeting their instructors for the first time and those who were already familiar with them from previous classes. Notably, the data reveal no significant differences in preference between students who were already acquainted with their instructors and those experiencing their first interaction with them.

In Table 4, a comparison of scores between students preferring virtual and traditional teaching methods was presented, highlighting gender differences and student satisfaction. Among students who preferred virtual teaching, there was no significant difference between males (mean score: 11.62 + 3.62) and females (mean score: 11.06 + 3.28) (*p* = 0.152). However, in the traditional teaching group, male preference was significantly higher than female preference, with mean scores of 12.04 + 4.38 and 10.13 + 4.55, respectively (*p* = 0.001). Regarding student satisfaction, senior students reported slightly higher satisfaction with virtual teaching methods compared to junior students (11.52 + 3.82 vs. 11.22 + 3.20), and this difference was not statistically significant (*p =* 0.443). However, the traditional classroom setting was highly preferred by the junior-year students with a significantly higher mean square of 11.87 + 4.43 compared to senior-year students (9.85 + 4.50) (*p*=.001).

## 4. Discussion

The impact of the COVID-19 pandemic has affected all sectors around the world, forcing all countries around the world to impose different measures to cope and contain the spread of the infection. One of the responses of the educational sector was the shift to online/virtual learning. This study compared students’ perceptions and satisfaction with virtual vs. traditional learning. Overall students perceived traditional learning as better, and this perception was affected by factors such as gender, first-time exposure to the instructor, as well as the level of the study year.

In the current study, students were more satisfied with traditional learning than virtual learning, similar to what was reported from Iraq [26], Turkey [27], India [28], Pakistan [29], Italy [30], and Taiwan [31]. Some of the advantages of traditional learning, as reported by the students, were the increased interaction, the use of different instructional methods, and the availability of the course materials. The sudden transition to virtual instructions may not have allowed for better preparation and training of instructors, and a failure to develop the proper teaching skills, therefore minimizing the quality standard of education. Another reason that can explain why dental students preferred traditional learning is that the dental profession is more skill-based, and for a better understanding of concepts, face-to-face discussions with instructors would be more comfortable than virtual learning [32]. As per the survey findings, virtual learning is less interactive and has a reduced quality of learning compared to traditional learning methods. Personal attention and student engagement are two critical matters facing virtual learning; therefore, it is important to incorporate a variety of student engagement tools such as Kahoot, Socrative, and other online tools that facilitate active learning. Finally, instructors should consider how to enrich the student experience, while providing an opportunity for all students to experience alternative learning methods, and for the broadening of student and instructors’ views of what is considered participation in the classroom.

Virtual learning faces additional challenges regarding assessment methods. The evidence suggests that virtual examinations impose a greater cognitive load on students compared to traditional paper-based assessments. Students must simultaneously demonstrate their course knowledge while managing technological navigation and adapting to the increased complexity inherent to virtual assessment formats [33].

On the other hand, the reported advantages of virtual learning, compared to in-class traditional learning, were increased self-directed learning in virtual classes, better control over the pace of one’s learning, and more reflections on learning in virtual classrooms, as well as improvements in time management skills. Previous studies have also indicated that students prefer e-learning due to accessibility, timetable, and flexibility when studying abroad [34,35]. In the current study, students agreed that virtual learning helped them understand the scientific material clearly, provided them with additional training and skills, and improved their self-reflection skills. If used effectively and purposefully, reflection facilitates ongoing personal and professional learning, and creates and develops practitioners capable of demonstrating their progression toward learning outcomes and required standards [36].

Success in online learning environments demands enhanced self-regulatory abilities from students, with these self-regulation skills being influenced by various personal and demographic factors, particularly gender [37]. In the current study, males preferred traditional learning more than virtual learning. There was inconsistency in the research results that investigated gender preferences of instructional method type. Some found that males adapt better to virtual learning and adopt more behavioral strategies than females to deal with their disorientation during virtual learning [38]. Others reported that females have better online communication and self-efficacy than males [39,40], while few countries reported no significant gender differences in e-learning outcomes [41]. E-learning is closely related to social contexts of genders rather than the gender itself, which may explain the controversial results from different countries.

The present survey also analyzed students’ responses according to their years of dental education. It was observed that preclinical (junior) students (3rd year and 4th year) preferred traditional learning more than clinical (senior) (5th year and 6th year), contradicting the findings by Gormley et al. [42], who reported that undergraduate medical students considered e-learning or virtual to be just as effective as other traditional methods. However, it was reported in many studies that students found virtual learning to be effective for learning theoretical subjects but not for clinical subjects [43], which resonates with our findings as the majority of the courses taught to preclinical/junior students are theoretical.

The success of virtual learning depends on many factors, which include the experience and attitudes of students with regard to technology as well as the interactive teaching styles of the instructor [44]. In this study, students who were exposed to the instructors before the shift to virtual learning preferred the traditional methods more. Traditional learning, if well planned, can engage students and encourage active participation and motivation [45], which students reported to be missing during the online classes [46]. Although the COVID-19 pandemic led to a sudden and unprepared shift to online learning, it would be beneficial for dental schools to provide instructors not only with intense training on the technical aspects of the virtual platform itself, but also on basic principles of instructional design for effective virtual delivery to promote student engagement and appropriate assessment methodology.

The preference for traditional learning among both preclinical and clinical dental students can be rationalized through several evidence-based factors. Dental education heavily relies on psychomotor skills development, which is better achieved through direct physical interaction and immediate feedback in traditional settings [19]. Traditional learning provides a real-time demonstration and supervision of clinical procedures, which are crucial for developing proper technique and clinical judgment [20]. Moreover, traditional classrooms facilitate immediate peer-to-peer interactions and faculty feedback, essential components for developing clinical reasoning skills [21]. This is particularly important as dental procedures often involve complex spatial and tactile components that are better demonstrated and practiced in person [22]. Additionally, direct interaction with peers, faculty, and patients in traditional settings contributes to the development of professional identity and communication skills, which are fundamental aspects of dental education [10,47]. These factors collectively explain why students at both preclinical and clinical levels show a stronger preference for traditional learning methods. This study has several limitations that should be considered. The cross-sectional design only allowed for the identification of associations rather than causal relationships. The sample was limited to undergraduate students from a single dental institution, which restricts the generalizability of findings. A more diverse and larger sample size would have provided more comprehensive insights. Additionally, the reliance on self-reported data increases the potential for recall bias in the responses. The questionnaire design included statements that could potentially introduce bias and limit the range of student responses. The lack of a direct comparison between virtual and traditional methods for identical course content may have affected the validity of the comparison. Additionally, the limited response options may not have captured the full spectrum of student perceptions. Also, this study only determined the perception and satisfaction of dental students, which were subjective in nature. Future studies should consider both subjective and objective outcome measures. We recommend developing questionnaires with neutral language, incorporating 5-point Likert scales for more nuanced responses, and including open-ended questions for detailed feedback. Furthermore, future research should focus on delivering identical content through both virtual and traditional methods, evaluating immediate student satisfaction after each teaching modality, and assessing learning outcomes using standardized measurements. Comparing different university education systems is also recommended.

## 5. Conclusions

The key findings of the current study are:

- A strong preference for traditional learning, especially among preclinical dental students.
- Male students showed a particular preference for traditional methods.
- Students face engagement challenges in virtual formats.
- Traditional learning was appreciated for interactive depth while virtual learning was valued for structure, self-directed learning, and time management skills.
- Virtual learning is a supplementary tool that cannot completely replace traditional classrooms.
- Implementing a combined approach utilizing the strengths of both traditional and virtual learning methods is highly recommended.

## Figures and Tables

**Table 1:** Overall response of the participants to virtual and traditional learning (n = 313).

Questionnaire Items	Yes	No	No Difference
	n (%)		
Virtual learning is more convenient than traditional classroom	120 (38.3)	164 (52.4)	29 (9.3)
I interact more with instructors in traditional classroom than virtual classroom	176 (56.2)	73 (23.3)	64 (20.4)
Virtual learning does not allow interaction between students	120 (38.3)	140 (44.7)	53 (16.9)
Traditional classroom allows the use of different instructional methods	192 (61.3)	49 (15.7)	72 (23)
I comprehend/understand course materials more through traditional classroom	171 (54.6)	66 (21.1)	76 (24.3)
The format of the course encourages more self-directed learning in virtual classes	190 (60.7)	47 (15)	76 (24.3)
I have better control over the pace of my own learning in virtual classroom course	180 (57.5)	74 (23.6)	59 (18.8)
Course intended outcomes are clearer in traditional classrooms	160 (51.1)	54 (17.3)	99 (31.6)
Course learning materials are easily provided in traditional classrooms than virtual	139 (44.4)	87 (27.8)	87 (27.8)
Virtual classrooms improved my time management skills	161 (51.4)	100 (31.9)	52 (16.6)
Virtual classrooms are only effective for non-clinical courses	197 (62.9)	82 (26.2)	34 (10.9)
Virtual classrooms encourage me to reflect more on my learning	153 (48.9)	72 (23)	88 (28.1)
Lectures outlines are more organized in traditional classrooms than virtual	84 (26.8)	69 (22)	160 (51.1)
It is difficult to be attentive all the time in virtual classrooms	184 (58.8)	78 (24.9)	51 (16.3)
Virtual classrooms lack non-verbal communications that hinder my understanding	158 (50.5)	81 (25.9)	74 (23.6)
Virtual OSPE will be better for me than traditional one	137 (43.8)	122 (39)	54 (17.3)
Virtual written assessment will be better for me than traditional one	104 (33.2)	144 (46)	65 (20.8)
My Grades for topics covered by virtual methods will be better than those covered in conventional classroom	82 (26.2)	96 (30.7)	135 (43.1)

**Table 2:** Advantages of online/virtual learning as reported by students (n = 313).

Advantages of Online/Virtual Learning	Response	Academic Year Level				*p*-Value
		3	4	5	6	
Virtual learning is more convenient than traditional classroom	Yes	12.8%	54.7%	44.3%	46.0%	0.001 *
	No	87.2%	45.3%	55.7%	54.0%	
Virtual classrooms improved my time management skills	Yes	37.2%	50.5%	57.4%	68.3%	0.001 *
	No	62.8%	49.5%	42.6%	31.7%	
Virtual classrooms are only effective for non-clinical courses	Yes	76.6%	37.9%	65.6%	77.8%	0.001 *
	No	23.4%	62.1%	34.4%	22.2%	
Virtual classrooms encourage me to reflect more on my learning	Yes	47.9%	54.7%	54.1%	36.5%	0.118
	No	52.1%	45.3%	45.9%	63.5%	
The format of the course encourages more self-directed learning in virtual classes	Yes	68.1%	56.8%	73.8%	42.9%	0.001 *
	No	31.9%	43.2%	26.2%	57.1%	
Virtual OSPE will be better for me than traditional one	Yes	26.6%	48.4%	50.8%	55.6%	0.001 *
	No	73.4%	51.6%	49.2%	44.4%	
Virtual written assessment will be better for me than traditional one	Yes	39.4%	32.6%	41.0%	17.5%	0.016 *
	No	60.6%	67.4%	59.0%	82.5%	
My Grades for topics covered by virtual methods will be better than those covered in conventional classroom	Yes	24.5%	23.2%	42.6%	17.5%	0.009 *
	No	75.5%	76.8%	57.4%	82.5%	
I have better control over the pace of my own learning in virtual classroom course	Yes	43.6%	61.1%	72.1%	58.7%	0.004 *
	No	56.4%	38.9%	27.9%	41.3%	

* Showing the significant at 0.05.

**Table 3:** Advantages of traditional learning as reported by students.

		Academic Year Level				
Advantages of Traditional Learning	Response	3	4	5	6	*p*-Value
I interact more with instructors in traditional classroom than virtual classroom	Yes	70.2%	57.9%	39.3%	49.2%	0.001 *
	No	29.8%	42.1%	60.7%	50.8%	
Virtual learning does don’t allow interaction between students	Yes	48.9%	36.8%	32.8%	30.2%	0.068
	No	51.1%	63.2%	67.2%	69.8%	
Traditional classroom allows the use of different instructional methods	Yes	72.3%	53.7%	49.2%	68.3%	0.007 *
	No	27.7%	46.3%	50.8%	31.7%	
I comprehend/understand course materials more through traditional classroom	Yes	66.0%	54.7%	44.3%	47.6%	0.033 *
	No	34.0%	45.3%	55.7%	52.4%	
Course intended outcomes are clearer in traditional classrooms	Yes	62.8%	53.7%	42.6%	38.1%	0.010 *
	No	37.2%	46.3%	57.4%	61.9%	
Course learning materials are easily provided in traditional classrooms than virtual	Yes	53.2%	51.6%	32.8%	31.7%	0.006 *
	No	46.8%	48.4%	67.2%	68.3%	
Lectures outlines are more organized in traditional classrooms than virtual	Yes	29.8%	33.7%	27.9%	11.1%	0.014 *
	No	70.2%	66.3%	72.1%	88.9%	
It is difficult to be attentive all the time in virtual classrooms	Yes	73.4%	56.8%	45.9%	52.4%	0.003 *
	No	26.6%	43.2%	54.1%	47.6%	
Virtual classrooms lack non-verbal communications that hinder my understanding	Yes	62.8%	50.5%	36.1%	46.0%	0.010 *
	No	37.2%	49.5%	63.9%	54.0%	

* Showing the significant at 0.05.

**Table 4:** Score comparisons.

Demographic	Response	Preferred Virtual	*p*-Value	Preferred Traditional	*p*-Value
Gender	Male	11.62 + 3.62	0.152	12.04 + 4.38	0.001*
	Female	11.06 + 3.28		10.13 + 4.55	
Year Level	Junior/Preclinical	11.22 + 3.20	0.443	11.87 + 4.43	0.001*
	Senior/Clinical	11.52		9.85 + 4.50	

* Showing the significance at 0.05.

## Data Availability

Data will be provided upon request from the corresponding author.
